# Lung function decline is associated with serum uric acid in Korean health screening individuals

**DOI:** 10.1038/s41598-021-89678-3

**Published:** 2021-05-13

**Authors:** Kyung-Min Ahn, Suh-Young Lee, So-Hee Lee, Sun-Sin Kim, Heung-Woo Park

**Affiliations:** 1 Department of Internal Medicine, Seoul National University Hospital, Seoul, Republic of Korea; 2 Seoul National University Hospital Healthcare System Gangnam Center, Seoul, Republic of Korea; 3 Department of Internal Medicine, Seoul National University College of Medicine, Seoul, Republic of Korea; 4 Institute of Allergy and Clinical Immunology, Seoul National University Medical Research Center, Seoul, Republic of Korea

**Keywords:** Biomarkers, Medical research, Risk factors

## Abstract

We performed a retrospective cohort study of 19,237 individuals who underwent at least three health screenings with follow-up periods of over 5 years to find a routinely checked serum marker that predicts lung function decline. Using linear regression models to analyze associations between the rate of decline in the forced expiratory volume in 1 s (FEV1) and the level of 10 serum markers (calcium, phosphorus, uric acid, total cholesterol, total protein, total bilirubin, alkaline phosphatase, aspartate aminotransferase, creatinine, and C-reactive protein) measured at two different times (at the first and third health screenings), we found that an increased uric acid level was significantly associated with an accelerated FEV1 decline (P = 0.0014 and P = 0.037, respectively) and reduced FEV1 predicted % (P = 0.0074 and P = 8.64 × 10^–7^, respectively) at both visits only in non-smoking individuals. In addition, we confirmed that accelerated forced vital capacity (FVC) and FEV1/FVC ratio declines were observed in non-smoking individuals with increased serum uric acid levels using linear mixed models. The serum uric acid level thus potentially predicts an acceleration in lung function decline in a non-smoking general population.

## Introduction

Lung function (LF) gradually declines over time with wide inter-individual variations^1,2^. Individuals exhibiting an accelerated LF decline are susceptible to chronic respiratory diseases and are at higher risk of all-cause mortality^3,4^. Therefore, the identification of biomarkers associated with such a decline would be of a great assistance^5^.

To gain proper insight on LF decline, a longitudinal study of LF is essential. However, any long-term observational study must deal with subject drop-out, missing values, and incomplete information on variables that affect LF decline. Thus, health screening data are of particular interest. Although selection bias is potentially in play, health screenings yield, long-term repetitive measurements (without missing data) obtained using uniform standard methods and comprehensive information on co-morbidities and confounders^6^.

Blood is readily sampled and yields information allowing clinicians to diagnose and manage diseases. Many serum markers have been reported to be linked to LF and LF decline including uric acid (UA)^7^, calcium^8^, cholesterol^9^, protein^10^, bilirubin^11^, liver enzyme [*e.g.* aspartate transaminase (AST)]^12^, alkaline phosphatase (ALP)^13^, creatinine (Cr)^14^, and high-sensitivity C-reactive protein (hsCRP)^15,16^. However, most previous studies focused on single markers and thus did not capture possible interactions among markers. In addition, single measurements are unreliable, as serum marker levels commonly change over time. Here, we sought serum markers predictive of LF decline and explored whether the predictions were consistent over time. We thus carefully analyzed blood and LF test results recorded in a high-quality health screening database.

## Methods

The study protocol was approved by the Institutional Review Board of Seoul National University Hospital (H-1601-080-734). The Board waived the need for informed participant consent. We adhered to all relevant guidelines and regulations pertaining to good medical practice, clinical research, bioethics and data protection.

### Study population

We selected the study population from the database of the Seoul National University Hospital Healthcare System Gangnam Center located in Seoul, the Republic of Korea. All individuals were self-recruited for routine health screening, usually funded by either private or national health insurance. The database spanned circa 15 years (October 2004–May 2019). Individuals who underwent at least three screenings with a follow-up period (between the first and last screenings) more than 5 years were included. We excluded individuals who self-reported any respiratory disease affecting LF or LF decline (asthma, chronic obstructive disease [COPD], and pulmonary fibrosis). A smoker was defined a subject with a smoking history of > 10 pack-years; this significantly predicts an accelerated decline in forced expiratory volume in 1 s (FEV1)^17^.

### Measurements

We recorded gender, age, body mass index (BMI), and smoking status. Based on previous reports^7–16^, we evaluated the serum levels of calcium, phosphorus, UA, total cholesterol, total protein, total bilirubin, ALP, AST, Cr, and CRP in terms of potential associations with LF decline. Venous blood samples were collected before 10 AM after a 12-h overnight fast. Spirometric LF measurements were performed by experienced technicians who followed the American Thoracic Society recommendations^18^. A flow-sensing spirometer was employed (MasterScreen PNEUMO, Viasys Respiratory Care, Inc., San Diego, CA, USA). The forced vital capacity (FVC) and FEV1 were measured and expressed as both absolute values (mL) and predicted values (%) calculated using formulae appropriate for ethnic Koreans^19^. All LF tests were done without bronchodilator inhalation.

### Statistical analysis

For each individual, the slope representing the FEV1 decline rate (mL/year) was calculated using all FEV1 measurements obtained over the follow-up period. We next performed multivariate linear regression to identify serum markers associated with the FEV1 decline rate. The regression model included age, gender, BMI, smoking status, the FEV1 predicted % value, and the levels of 10 serum markers measured at the first health screening (T1). To assess data consistency, the analysis was repeated using the same variables measured at the third health screening (T2). We performed cross-sectional analyses using the FEV1 predicted % value measured at T1 or T2, as the dependent variable. We chose this parameter because participant height is employed in the calculation of FEV1 predicted % value^19^, and height is significant in terms of LF. We thought that these additional analysis helped us to find the serum markers more relevant to LF decline. Statistical analyses were performed with R version 4.0.2. software. Two-sided *P* values < 0.05 were considered indicative of statistical significance. To exclude any possible multicollinearity among independent variables, we applied a variance inflation factor to the final model. Finally, to confirm our observation, we used linear mixed models for the association between serum markers identified and LF measurements (FEV1, FVC and FEV1/FVC ratio) adjusting for age, gender, height, and BMI at T1. Random effects were used to account for the correlation between repeated measures within each individual.

## Results

**Figure 1:**
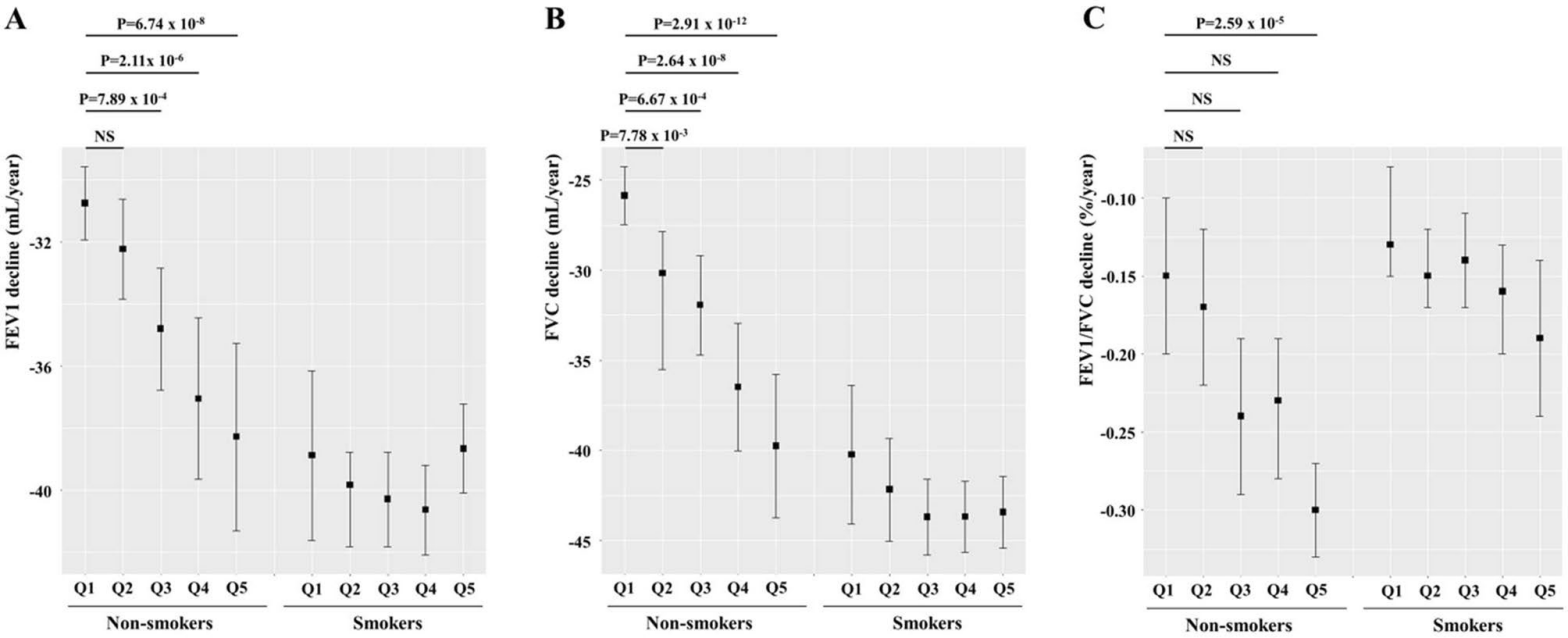
Lung function decline in individuals (non-smokers and smokers) stratified according to serum uric acid quintile. (**A**) FEV1, (**B**) FVC, (**C**) FEV1/FVC ratio. Each bar represents mean with 95% confidence interval. The serum uric acid quintile groups were determined based on measurements performed at T1. The mean serum uric acid levels (standard deviations) for the quintiles were as follows: Q1 3.96 (0.73) mg/dL, Q2 4.77 (0.65) mg/dL, Q3 5.43 (0.79) mg/dL, Q4 6.18 (0.78) mg/dL, and Q5 7.12 (1.13) mg/dL. No significant difference in FEV1, FVC, and FEV1/FVC ratio declines were evident between smokers in Q1 and those in the other quintiles. NS, not significant. This graph was generated using R Software 4. 0. 2. [R Core Team (2020). R: A language and environment for statistical computing. R Foundation for Statistical Computing, Vienna, Austria. URL https://www.R-project.org/].

A total of 35,129 individuals were listed in the database. Of these, 19,237 who met our enrollment criteria were included. There were 11,258 (58.5%) males, and 9,860 individuals (51.3%) were non-smokers. The average follow-up duration (between T1 and the last health screening) was 8.95 years [standard deviation (SD), 2.55; range, 5–14.5 years)] and the average number of health screening was 6.51 (SD, 2.82; range, 3–16). The average FEV1 decline rate was -37.32 mL/year (SD, 12.23; range  − 0.01 ~  − 144.72). Individual characteristics at T1 (the first health screening) and T2 (the third screening) are summarized in Table 1. The average gap between T1 and T2 was 4.05 years (SD, 2.42; range, 1–14.4 years). Table 2 shows the results of multivariate regression analysis of the FEV1 decline rate using the measurement obtained at T1 and T2. As expected, male sex and smoking status were significantly associated with greater FEV1 declines. In terms of serum markers, an increased uric acid level was significantly associated with an accelerated FEV1 decline rate at both T1 (*P* = 0.0014) and T2 (*P* = 0.0366); consistency was thus evident (Figure S1). Increased serum ALP and Cr levels were significantly associated with a decrease in FEV1 decline rates at both T1 and T2. Table 3 shows the FEV1 predicted % values at T1 and T2. Again, the serum uric acid was significantly (negatively) associated with the FEV1 predicted % at both T1 (*P* = 0.0074) and T2 (*P* = 8.64 × 10^–7^) Thus, we focused on the uric acid data. Uric acid is thought be an antioxidant; the level rises when oxidative stress caused by various stimuli increases. Such stressors include air pollution and smoking^20^. Smoking is a potent predictor of LF decline^21,22^. We thus divided all individuals into smokers and non-smokers and separately evaluated the effect of serum uric acid level at T1 and T2 on the FEV1 decline rate (Table 4). Notably, a significant association between the serum uric acid levels level and FEV1 decline rate was found only in non-smokers (at both T1 and T2) (Table 4). Similarly, linear mixed regression analysis revealed that the serum uric acid level was significantly associated with a greater FEV1, FVC, and FFV1/FVC ratio decline only in non-smokers (*P* = 9.28 × 10^–6^, *P* = 3.04 × 10^–7^, and *P* = 2.25 × 10^–4^, respectively). We divided all individuals into five groups according to quintiles of serum uric acid measurements at T1 and repeated the linear mixed regression analysis. Interestingly, non-smoking individuals in the third [mean, − 34.80 mL/year; 95% confidence interval (CI), − 36.78 to − 32.85 mL/year], the fourth (mean, − 37.05 mL/year; 95% CI, − 39.64 to − 34.45 mL/year), and the fifth (mean, − 38.28 mL/year; 95% CI, − 41.31 to − 35.26 mL/year) quintiles exhibited significantly (dose-dependent) slower FEV1 declines compared to non-smoking individuals in the first quintile (mean, − 30.76 mL/year; 95% CI, − 31.94 to − 29.58 mL/year) (Fig. 1A). For FVC decline, the same trend is observed in non-smoking individuals (Fig. 1B). Meanwhile, only non-smoking individuals in the fifth quintile showed a significantly accelerated FEV1/FVC decline compared to non-smoking individuals in the first quintile (Fig. 1C).

**Table 1:** General characteristics and laboratory parameters of the enrolled individuals at the first and third health screenings.

Variables	1st health screening	3rd health screening
Age (year)	45.56 ± 10.17	49.62 ± 9.93
Body mass index (Kg/m^2^)	23.42 ± 16.11	23.32 ± 3.07
FEV1 (mL)	3172.99 ± 685.65	3059.45 ± 1171.09
FEV1 predicted (%)	103.34 ± 12.69	103.16 ± 13.24
FVC (mL)	3862.08 ± 838.34	3772.74 ± 832.42
FVC predicted (%)	95.46 ± 11.16	95.49 ± 11.34
FEV1/FVC (%)	82.49 ± 6.83	81.06 ± 6.46
Calcium (mg/dL)	9.24 ± 0.38	9.14 ± 0.36
Phosphorus (mg/dL)	3.60 ± 0.49	3.53 ± 0.50
Uric acid (mg/dL)	5.51 ± 1.46	5.43 ± 1.42
Total cholesterol (mg/dL)	194.20 ± 33.35	193.82 ± 33.01
Total protein (g/dL)	7.18 ± 0.41	7.14 ± 1.02
Total bilirubin (mg/dL)	1.09 ± 0.42	1.00 ± 0.39
Alkaline phosphatase (unit/L)	58.17 ± 16.50	54.67 ± 15.57
Aspartate transaminase (unit/L)	23.69 ± 13.71	23.64 ± 13.39
Creatinine (mg/dL)	0.98 ± 0.20	0.89 ± 0.32
C-reactive protein (mg/L)	0.12 ± 0.34	0.12 ± 0.37

Data present presented as mean ± standard deviation.

**Table 2:** Associations between clinical and serum parameters measured at the first and third health screenings and the rate of FEV1 decline.

Variables	1st health screening		3rd health screening	
	Beta	*P* value	Beta	*P* value
Male	− 5.652	** < 2E–16**	− 5.416	** < 2E–16**
Age (year)	− 0.142	** < 2E–16**	− 0.151	** < 2E–16**
Body mass index (Kg/m^2^)	0.007	0.1716	0.072	0.0559
Smoking status (Yes)	− 0.853	**0.0010**	− 0.496	**0.0175**
FEV1 predicted %	− 0.222	** < 2E–16**	− **0.019**	**0.0301**
Calcium (mg/dL)	− 0.519	0.1021	− 0.458	0.1709
Phosphorus (mg/dL)	0.658	**0.0017**	0.207	0.3353
Uric acid (mg/dL)	− 0.288	**0.0014**	− 0.179	**0.0366**
Total cholesterol(mg/dL)	0.001	0.8499	− 0.002	0.5601
Total protein (g/dL)	− 0.014	0.9600	0.959	**0.0002**
Total bilirubin (mg/dL)	− 0.115	0.6274	0.014	0.9596
Alkaline phosphatase (unit/L)	0.053	** < 2E–16**	0.033	**1.35E–06**
Aspartate transaminase (unit/L)	0.007	0.3727	− 0.015	0.0591
Log_2_(Creatinine) (mg/dL)	8.214	** < 2E–16**	5.256	** < 2E–16**
C-reactive protein (mg/L)	− 0.023	0.9364	− 0.306	0.2535

Bolds represent significant results (*P* < 0.05).

**Table 3:** Associations between clinical and serum parameters and FEV1% predicted value at the first and third health screenings.

Variables	1st health screening		3rd health screening	
	Beta	*P* value	Beta	*P* value
Male	− 0.892	**0.0228**	− 2.614	**4.13E–11**
Age (year)	0.193	** < 2E–16**	0.184	** < 2E–16**
Body mass index (Kg/m^2^)	0.004	0.4595	0.290	**8.08E–13**
Smoking status (Yes)	− 1.265	**1.02E–05**	− 1.156	**4.40E–05**
Calcium (mg/dL)	1.397	**6.69E–05**	1.906	**1.40E–07**
Phosphorus (mg/dL)	− 0.074	0.7498	0.630	**0.0067**
Uric acid (mg/dL)	− 0.267	**0.0074**	− 0.501	**8.64E–07**
Total cholesterol (mg/dL)	0.004	0.1922	0.008	**0.0176**
Total protein (g/dL)	− 0.769	**0.0098**	− 1.385	**7.52E–07**
Total bilirubin (mg/dL)	1.238	**2.01E–06**	1.774	**1.19E–09**
Alkaline phosphatase (unit/dL)	− 0.026	**0.0002**	− 0.014	0.0611
Aspartate transaminase (unit/dL)	− 0.020	**0.0229**	− 0.019	**0.0231**
Log2(Creatinine) (mg/dL)	− 0.670	0.1967	2.719	**1.64E–08**
C-reactive protein (mg/L)	− 1.568	**8.01E–07**	− 1.025	**4.26E–04**

Bolds represent significant results (*P* < 0.05).

**Table 4:** Associations between clinical and serum parameters and FEV1 decline rate at the first and third health screenings by smoking status.

Variables	Non-smoker				Smoker			
	1st health screening		3rd health screening		1st health screening		3rd health screening	
	Beta	*P* value	Beta	*P* value	Beta	*P* value	Beta	*P* value
Male	− 5.642	** < 2E–16**	− 4.557	** < 2E–16**	− 5.629	** < 2E–16**	− 6.653	** < 2E–16**
Age (year)	− 0.151	** < 2E–16**	− 0.176	** < 2E–16**	− 0.159	** < 2E–16**	− 0.130	**2.93E–16**
Body mass index	0.321	**1.90E–10**	0.091	6.53E–02	0.004	0.495	0.067	0.240
FEV1 predicted %	− 0.219	** < 2E–16**	0.031	**1.87E–03**	− 0.236	** < 2E–16**	0.007	0.543
Calcium (mg/dL)	0.498	0.214	1.046	**0.016**	− 1.644	**1.05E–03**	− 2.019	**8.17E–05**
Phosphorus (mg/dL)	0.716	**7.86E–03**	0.088	0.750	0.664	**4.29E–02**	0.423	0.206
Uric acid (mg/dL)	− 0.640	**1.62E–06**	− 0.256	**0.048**	− 0.197	0.121	− 0.120	0.362
Total cholesterol (mg/dL)	− 0.001	0.821	− 0.002	0.548	− 0.001	0.852	0.001	0.990
Total protein (g/dL)	− 0.904	**0.009**	0.142	0.674	1.055	**0.012**	1.709	**1.55E–05**
Total bilirubin (mg/dL)	− 0.187	0.556	0.200	0.594	0.068	0.847	− 0.145	0.708
Alkaline phosphatase (unit/dL)	0.054	**1.66E–11**	0.034	**7.97E–05**	0.043	**1.29E–05**	0.033	**1.87E–03**
Aspartate transaminase (unit/dL)	0.007	0.538	− 0.019	0.153	0.002	0.841	− 0.012	0.226
Log2(Serum creatinine) (mg/dL)	7.568	** < 2E–16**	3.339	**2.66E–09**	8.828	** < 2E–16**	7.543	** < 2E–16**
C-reactive protein (mg/L)	0.671	0.145	− 0.218	0.596	− 0.507	0.183	− 0.387	0.283

Bolds represent significant results (*P* < 0.05).

## Discussion

Using annual health screening data on 19,237 individuals, we found that increased serum uric acid levels measured at two different times were consistently and significantly associated with accelerated FEV1 decline and reduced FEV1 predicted % values in non-smoking individuals. In addition, we confirmed that accelerated FVC and FEV1/FVC ratio declines were observed in non-smoking individuals with increased serum uric acid levels using linear mixed analysis. As serum uric acid levels are relatively stable over time^23^, the uric acid level may serve as a simple but valuable predictor of LF decline in clinical practice, especially in non-smokers. To the best of our knowledge, this is the first longitudinal analysis of an association between the serum uric acid level and LF decline.

As mentioned above, several serum markers have been reported to be related to LF and LF decline. However, no single marker can capture all interactions among serum markers; any perceived association may thus be. For example, obesity is frequently associated with a fatty liver, dyslipidemia, and gout; close correlations are hence apparent between serum total cholesterol, AST, ALT, and uric acid levels. It is thus necessary to consider possible interactions when evaluating the effects of serum markers on LF or LF decline. In addition, serum marker levels usually change over time^24^. If a marker is to be clinically useful, the level must remain constant over time We found that the serum uric acid level usefully predicted the FEV1 decline rate after adjusting for a further 9 serum markers and several clinical variables. In addition, significant associations were evident at both the first and third screenings, separated by approximately 4 years. The levels, at both times were closely correlated (R^2^ square = 0.809, *P* < 2.0 × 10^–16^, Figure S1). When evaluating LF decline, long-term observations using a full set of relevant are essential. The health-screening database was a good source of data; it contains comprehensive long-term repeated measurements collected employing uniform standard methods. We enrolled a large number of individuals with relatively long follow-up periods during which several measurements were made. We thus suggested that our results are more reliable than those of previous studies.

Any role for uric acid in inflammation remains controversial. Uric acid serves as a potent upper airway antioxidant, scavenging peroxynitrite and eliminating reactive oxygen species (ROS)^25^. Uric acid is an important first-line defense against ROS and is present in at high concentrations in the epithelial fluid of the human respiratory tract^26,27^. Uric acid also serves as a pro-inflammatory molecule in endothelia^28,29^. Recently, the serum uric acid level has been suggested to serve as a marker of respiratory disorders including COPD^30,31^, asthma^32^, obstructive sleep apnea^33^, pulmonary hypertension^34^, and chronic respiratory failure^35^. The uric acid level reflects the extents of tissue hypoxia and systemic inflammation. The negative association between the serum uric acid level and LF is open to several possible explanations. First, inflammation triggered by uric acid may be associated with the proliferation and activation of inflammatory cells of the respiratory epithelial lining; the activities of pro-inflammatory pathways then increase^36,37^. Over the last decade, associations between hyperuricemia and all of cardiovascular diseases, renal interstitial inflammation, and tubular injury have been reported^38,39^. One possible mechanism by which uric acid triggers inflammation involves the profibrotic factor endothein-1^40^. This potent vasoconstrictor is elevated not only in patients with vascular dysfunctions, such as myocardial infarction, stroke, and hypertension, but also in those with respiratory diseases, asthma, and COPD. Known as an up-regulator of other inflammatory mediators^41^, IL-6 and IL-8, endothelin-1 has been shown to stimulate mucus production, airway edema, and bronchial hyper-responsiveness^42^. Uric acid stimulates the expression of endothelin-1^43,44^. Second, an elevated uric acid a level may reflect a comorbidity. A considerable proportion of patients with chronic airway obstruction also suffer from comorbid, systemic inflammatory diseases, such as cardiovascular disease, gout, or metabolic syndrome^45^. It is thus possible that an underlying condition may increase uric acid production. Third, hypoxia caused by impaired LF may induce excess uric acid production^31,46^. In several prior studies, an elevated serum uric acid level was correlated with the exacerbation of COPD and disease-related mortality^7,47–49^. The serum uric acid level was high especially in individuals exhibiting severe airflow limitations and frequent COPD exacerbations^49^. Tissue hypoxia and impaired oxidative metabolism can trigger purine catabolic pathways^47^, thus causing uric acid overproduction^7^. In several hypoxic scenarios, serum uric acid levels were consistently associated with serious hypoxia^49^. In our present study, however, most participants exhibited preserved average FEV1 values (103.34 ± 12.69 mL at the first visit and 103.16 ± 13.24 mL at the third visit). Fourth, after prolonged hypoxemia, the increased right ventricle afterload may cause excessive uric acid synthesis. Hyperuricemia has been reported in patients with severe pulmonary hypertension. Tissue ischemia reduces the ATP level, thus activating xanthine oxidase and increasing uric acid formation^49^.

If uric acid truly contributes to LF decline, uric acid-lowering agents may be protective. Several clinical studies have explored whether such agents improve LF. The hypoxanthine analog allopurinol dramatically improved the FEV1 in patients who received lung transplantation^50^, and also enhanced the six-minute walk test and COPD Assessment Test scores of COPD patients^51^. Allopurinol may reduce oxidative stress. In addition, febuxostat, a xanthine oxidase inhibitor, protected rats against lung injury caused by exposure to a respiratory irritant^52^.

Notably, we found a significant association between the serum uric acid level and the FEV1 decline rate in only non-smokers (Fig. 1 and Table 4). The correlations between the FEV1 decline rate and the serum uric acid level at T1 and at T3 were similar (Figure S2). Horsfall, et al. used the electronic database of a primary care clinic to explore the relationships between serum uric acid levels and respiratory diseases according to smoking status^53^. The cited authors found positive correlations between the serum uric acid level and incidences of respiratory diseases, COPD, and lung cancer, in both non-smokers and ex-smokers. Unlike current smokers, non-smoking and ex-smoking participants exhibited higher rates of COPD and lung cancer as the serum uric acid level rose. Although the differences did not attain statistical significance, the data indicate that uric acid may play a pro-inflammatory role in terms of LF. In another cross-sectional study, significant negative correlations were observed between the serum uric acid level and LF in both male and female^54^. The pro-inflammatory effects of uric acid may compromise the LF of non-smokers, as discussed above. Smoking powerfully affects LF, perhaps, masking any effect of uric acid on LF decline. Further studies are needed.

The serum Cr and ALP levels were significantly (negatively) associated with the FEV1 decline rate, but not the predicted FEV1% values. Cr and ALP may protect against FEV1 decline. Serum Cr is a metabolite of creatinine phosphate used by muscle cells^55^ as an energy source and is thus also a precursor of that energy source^56^. Recently, several studies have shown that Cr plays important metabolic roles and that the serum Cr level may reflect bodily muscle mass in a manner independent of the estimated glomerular filtration rate^57,58^. In our individuals higher serum Cr levels reflected bulkier muscle mass rather than poor renal function. A high muscle mass may protect against FEV1 decline^59,60^. However, the association between the serum ALP level and LF decline remains unclear. We postulate that ALP plays roles in musculoskeletal homeostasis and tissue damage. However, further evidence of a link between the ALP and LF decline is required.

Our work has several limitations. Firstly, although we tried to exclude individuals with respiratory diseases based on health screening questionnaires and chest X-ray (or chest computed tomography), we might include individuals with “hidden” asthma or COPD as only pre-bronchodilator FEV1 values were available. However, individuals who reported respiratory diseases as co-morbidities at T1 or T3 were excluded, and only 3.65% of all individuals had FEV1/FVC ratios of < 0.7 at T1. Thus, the risk of (inadvertent) inclusion of asthma or COPD patients was low. Secondly, as individuals chose to engage in annual, routine health screening, selection bias might have been in play. In addition, as we did not exclude individuals with non-respiratory co-morbidities (such as hypertension and diabetes mellitus), the individuals enrolled might thus not be representative of a normal healthy aging population. Thirdly, other LF parameters, such as peak expiratory flow, diffusion capacity or residual volume were not available in health screening database and this point should be considered when our observations are generalized. Finally, we lack molecular data on how the serum uric acid level affects FEV1 decline.

In conclusion, we found that increased serum uric acid level was significantly associated with accelerated FEV1, FVC, and FEV1/FVC declines in non-smoking individuals. Therefore, serum uric acid may be used to predict LF decline in a general population.

## Supplementary Information

Supplementary Information.

## Abbreviations

FEV1: Forced expiratory volume in 1 s
FVC: Forced vital capacity
LF: Lung function
