# Supplementary material for: Lung function decline is associated with serum uric acid in Korean health screening individuals
Source: Sci Rep. 2021 May 13;11:10183. doi: 10.1038/s41598-021-89678-3 (PMC8119944; doi:10.1038/s41598-021-89678-3)
Supplement: Supplementary file 1 — Supplementary Information. [file 41598_2021_89678_MOESM1_ESM.pdf]

Supplementary information

Lung function decline is associated with serum uric acid in Korean health screening individuals

Kyung-Min Ahn, MD[1], Suh-Young Lee, MD,[1] So-Hee Lee, MD,[1,2] Sun-Sin Kim, MD, PhD[1,2,*] Heung-Woo Park, MD, PhD[1,3,4*]

[1]Department of Internal Medicine, Seoul National University Hospital, Seoul, Republic of Korea

[2]Seoul National University Hospital Healthcare System Gangnam Center, Seoul, Republic of Korea

[3]Department of Internal Medicine, Seoul National University College of Medicine, Seoul, Republic of Korea

[4]Institute of Allergy and Clinical Immunology, Seoul National University Medical Research Center, Seoul, Republic of Korea

FIGURE LEGENDS

Figure S1. Correlation of serum uric acid between the first and second health screening

Figure S2. Simple correlations between serum uric acid levels and FEV1 decline rates at the first and third health screening according to smoking status.

A. Whole individuals at the first health screening

B. Smokers (green) and non-smokers (red) at the first health screening

C. Whole individuals at the third health screening

D. Smokers (green) and non-smokers (red) at the first health screening

Figure S1.

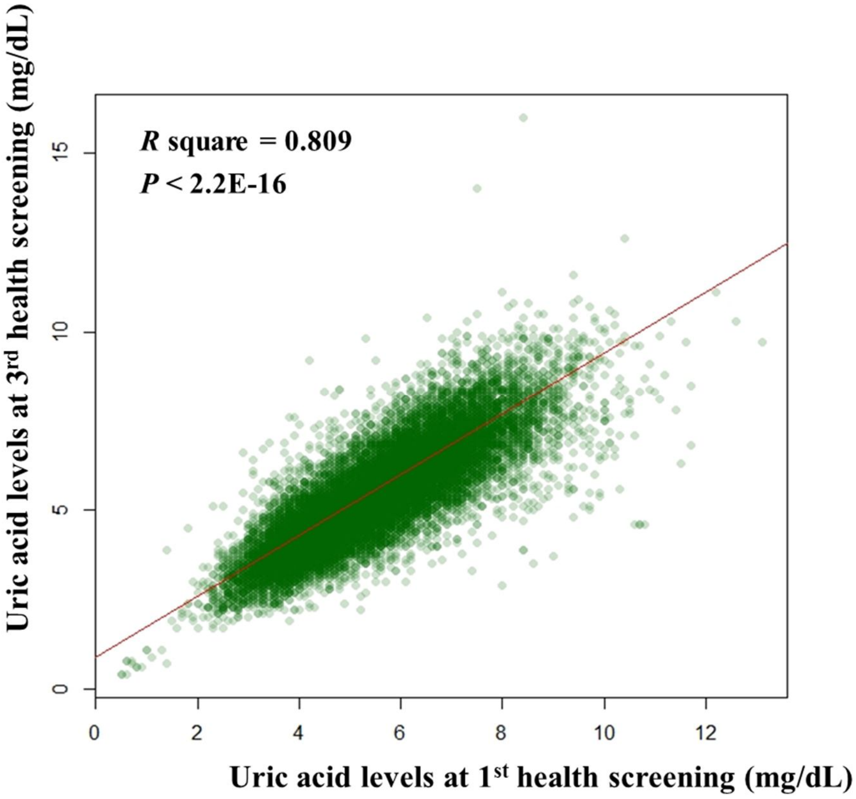

R square = 0.809
P < 2.2E-16
Uric acid levels at 3rd health screening (mg/dL)
Uric acid levels at 1st health screening (mg/dL)
0
5
10
15
0
2
4
6
8
10
12

Figure S2

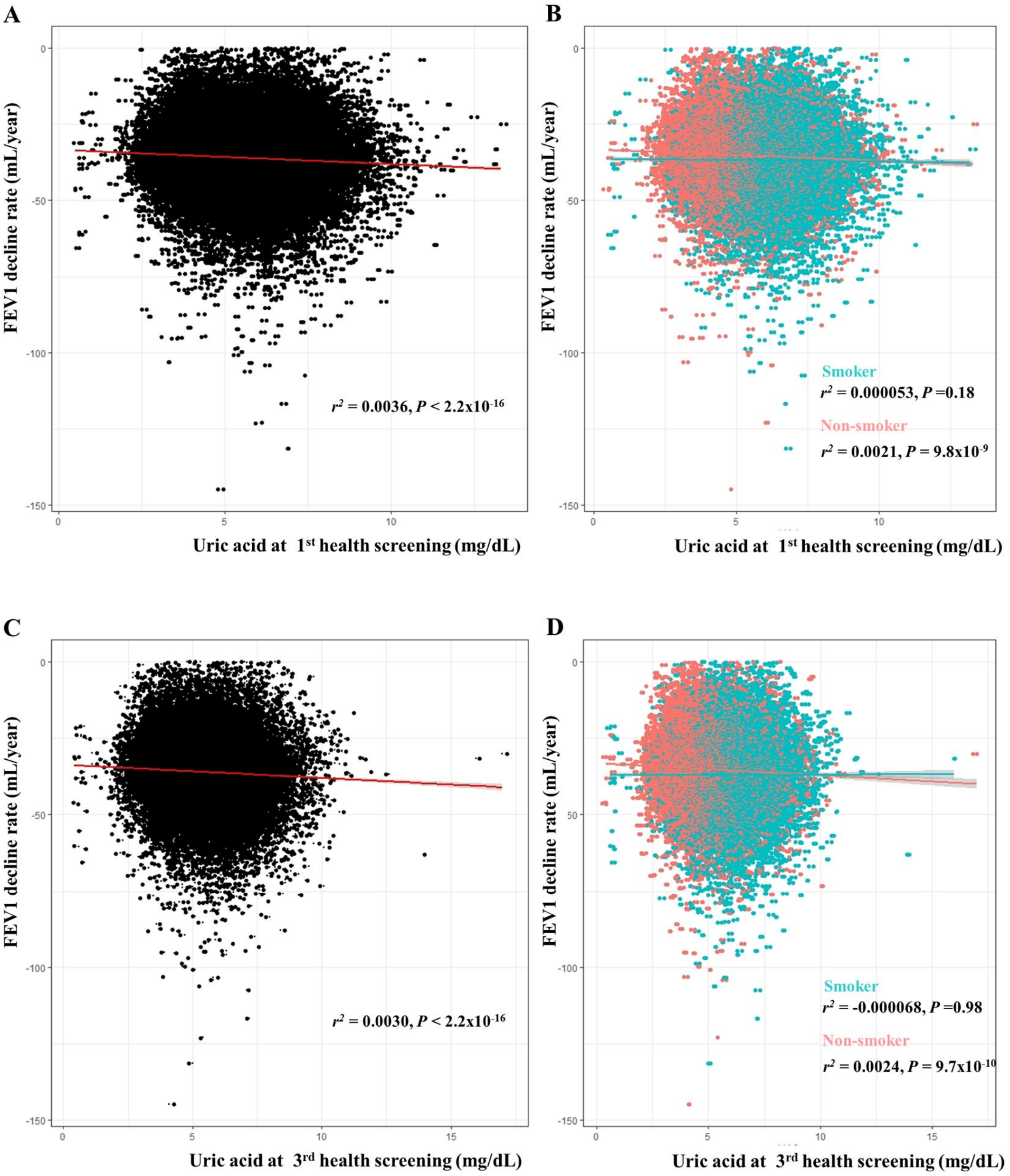
A
B
C
D
FEV1 decline rate (mL/year)
Uric acid at 1st health screening (mg/dL)
Uric acid at 3rd health screening (mg/dL)
r^2 = 0.0036, P < 2.2x10^{-16}
Smoker
r^2 = 0.000053, P =0.18
Non-smoker
r^2 = 0.0021, P = 9.8x10^{-9}
r^2 = 0.0030, P < 2.2x10^{-16}
Smoker
r^2 = -0.000068, P =0.98
Non-smoker
r^2 = 0.0024, P = 9.7x10^{-10}
